# Authors' reply

**DOI:** 10.4103/0301-4738.58485

**Published:** 2010

**Authors:** Sanjiv Kumar Gupta, Ajay Kumar, Deepak Kumar, Swati Agarwal

**Affiliations:** Department of Ophthalmology (SKG, DK, SA), KGMU, Lucknow; Jankalyan Eye Hospital (AK), Lucknow, India

Dear Editor,

I thank Chandravanshi for their keen interest and observations on our article.[1] My explanation for the issues raised is as follows. I hope this answers the issues raised by the reader.

- The apprehensive and uncooperative attitude was not a criterion for exclusion. One needs to understand that these are individuals who were capable of understanding and could follow commands; it was just that they were unaware of the procedure and unsure about the experience with more than average sensitivity. We did have such patients and they were managed by a little persuasion and firm attitude. The involuntary unwanted ocular movements may be a relative contraindication as this will make the procedure difficult.
- We have indicated the dosage of the intracameral injection of 0.5% lignocaine solution in the article, that this solution was injected only once (Materials and methods).[1] Comment on the safety of intracameral lignocaine with preservatives was based on the postoperative clinical picture and final outcome. We have not measured the endothelial cell count before or after the surgery.
- The lignocaine jelly 2% was not washed off before starting the surgery, it was allowed to come in contact with the scleral surface after conjunctival periotomy was done (to aid anesthesia). Sometimes in deep-set eyes the collection of jelly was excessive, in these situations we wiped the jelly off the surface. The possibility of jelly entering the anterior chamber may be hypothesized but we must keep in mind that modern cataract surgery is a positive pressure surgery, where we keep the chamber inflated, either by using intra cameral viscoelastics, or balanced salt solution, thus flushing out of the contents from the anterior chamber is expected rather than ingress of surrounding fluid inside the anterior chamber. The referred article about the relation between topical anesthesia and risk of endophthalmitis is misleading[2] as the risk is not related to topical anesthesia but rather to clear corneal incisions as reported by various authors.[3,4] I would like to add that all the preparations of lignocaine jelly may not be suitable for use on ocular surface; we used xylocaine 2% from Astra Zeneca India. This is autoclaved before use and one tube is used for all the patients in an operation theatre. Using a separate tube for each patient may be a preferred method of excluding inter-patient contamination.
- Regarding the raised intraocular pressure (IOP) induced due to peribulbar or retrobulbar injections, I must emphasize that there is universal rise in IOP after the administration of retrobulbar or peribulbar anesthesia, and this rise may be as high as more than 50 mm of Hg.[5] It is this rise in IOP for which we need ocupressure to redistribute the anesthetic agent and to increase the aqueous outflow to bring the IOP down so that the globe may be entered without any risk of positive vitreous pressure or expulsive hemorrhage. One must also understand the difference between the raised IOP before entering the anterior chamber and the raised pressure due to anterior chamber maintainer, viscoelastic substance, and infusion canula during pars plana vitrectomy. The risk of positive vitreous pressure, shallow anterior chamber, expulsive hemorrhage is because of sudden decompression of the anterior chamber due to differential pressure in the anterior chamber and posterior segment wherein contents from the posterior segment tend to prolapse into the anterior chamber. Maintaining and increasing the IOP during the surgery does not involve sudden release of pressure and thus there is no risk of positive vitreous pressure, or expulsive hemorrhage. Blepharospasm and lid squeezing are both due to inadequate anesthesia and pain. We have shown in the study that 95% patients had a pain evaluation of less than 3 on visual analog scales (threshold of significant pain).[1] Thus these factors do not come into play.
